# NABP-BERT: NANOBODY®-antigen binding prediction based on bidirectional encoder representations from transformers (BERT) architecture

**DOI:** 10.1093/bib/bbae518

**Published:** 2024-12-17

**Authors:** Fatma S Ahmed, Saleh Aly, Xiangrong Liu

**Affiliations:** Department of Computer Science and Technology, Xiamen University, Xiamen 361005, China; Department of Electrical Engineering, Aswan University, Aswan 81542, Egypt; Department of Information Technology, Majmaah University, Majmaah 11952, Saudi Arabia; Department of Computer Science and Technology, Xiamen University, Xiamen 361005, China

**Keywords:** NANOBODY®, antigen, BERT, binding prediction, deep learning, sequence embedding

## Abstract

Antibody-mediated immunity is crucial in the vertebrate immune system. Nanobodies, also known as VHH or single-domain antibodies (sdAbs), are emerging as promising alternatives to full-length antibodies due to their compact size, precise target selectivity, and stability. However, the limited availability of nanobodies (Nbs) for numerous antigens (Ags) presents a significant obstacle to their widespread application. Understanding the interactions between Nbs and Ags is essential for enhancing their binding affinities and specificities. Experimental identification of these interactions is often costly and time-intensive. To address this issue, we introduce NABP-BERT, a deep-learning model based on the BERT architecture, designed to predict NANOBODY®-Ag binding solely from sequence information. Furthermore, we have developed a general pretrained model with transfer capabilities suitable for protein-related tasks, including protein-protein interaction tasks. NABP-BERT focuses on the surrounding amino acid contexts and outperforms existing methods, achieving an AUROC of 0.986 and an AUPR of 0.985.

## Introduction

Antibodies (Abs) are proteins that recognize and bind to specific molecular sites on antigens (Ags), potentially harmful molecules, to trigger an immune response [1]. Due to their versatile binding capabilities, antibodies represent the predominant category of biotherapeutics, with 6 out of the top 10 most successful drugs being antibodies and a market value exceeding 100 billion dollars. The clinical development of antibody-based medicines is complex and time-consuming, often spanning several years [2, 3]. Abs are complex molecules composed of two polypeptide chains that require simultaneous expression and engineering, which can pose significant challenges. Due to their substantial size, the protein delivery might be challenging, especially in difficult situations such as tumor penetration. Therefore, there is considerable interest in exploring alternative Ab formats with more favorable therapeutic attributes. A subclass of Abs, called nanobodies (Nbs) or single-domain antibodies (sdAbs), has been discovered in camelid species such as camels, llamas, and alpacas [4].

Nbs exhibit structural similarities to normal Abs, but their Ag-binding regions are composed of a single polypeptide chain without a light chain. Nbs possess superior biophysical and therapeutic characteristics compared to Abs due to their smaller size (12$\sim $15 kDa) while still maintaining the benefits of molecular recognition [5] and possessing enhanced thermal stability. Furthermore, observations have shown that Nbs can identify hidden epitopes, penetrate regions inaccessible to regular Abs, and exhibit greater longevity and solubility compared to conventional Abs [4, 6]. Due to these beneficial pharmacological characteristics, Nbs are highly suitable for systematically developing multi-component medicines. Integrating Nbs created using traditional laboratory techniques into clinical settings will require several years. To overcome this challenge, computational approaches can expedite the process, thereby enhancing the accessibility and affordability of life-saving treatments.

Pretrained models, such as Bidirectional Encoder Representations from Transformers (BERT) [7] and GPT [8], have recently achieved significant success and are regarded as major advancements in artificial intelligence [9]. These High-capacity, self-supervised pretrained models can effectively learn from extensive datasets due to their numerous parameters. The vast knowledge embedded in these parameters can be leveraged to enhance various downstream tasks by finetuning these models for specific tasks. This approach, which utilizes extensive protein sequence data, has significantly improved the accuracy of predicting protein functions.

This study presents PROT-BERT, a specialized self-supervised pretrained model designed to learn detailed representations of general protein sequences. PROT-BERT can serve as a foundation for various protein-related tasks. Furthermore, we introduce PPI-PROT-BERT, a supervised model trained to predict interactions between pairs of protein sequences. PPI-PROT-BERT is based on PROT-BERT and is a foundation for downstream Protein-Protein Interaction (PPI) tasks. Building on these models, we introduce the NANOBODY®-Antigen Binding Prediction model, named NABP-BERT, which is based on the pretrained BERT model. NABP-BERT is a novel supervised model that predicts the binding between pairs of nanobody-antigen (Nb-Ag) sequences. NABP-BERT is available in two variants: NABP-PROT-BERT and NABP-PPI-PROT-BERT, which utilize PROT-BERT and PPI-PROT-BERT as their pretrained models, respectively.

To our knowledge, NABP-BERT represents the first attempt to predict Nb-Ag binding using the BERT model. Our main contributions include (i) Presenting PROT-BERT, a self-supervised model for protein-related tasks. (ii) Introducing PPI-PROT-BERT, a supervised model for protein-protein interaction prediction tasks. (iii) Proposing NABP-PROT-BERT and NABP-PPI-PROT-BERT, two supervised models to predict binding between Nb-Ag pairs. (iv) Demonstrating that NABP-BERT models outperform existing state-of-the-art methods.

## Related works

Deep learning algorithms have successfully addressed several challenges in protein research, including structure prediction [10], function prediction [11], and binding site prediction [12]. Massively parallel sequencing technology has facilitated the generation of substantial volumes of Ab repertoire sequencing data, increasing the utilization of deep learning in Abs [13–15].

Predicting Ab–Ag interactions is a crucial aspect of immunology research, as it facilitates the development of enhanced treatments such as vaccines. Numerous systems and methods are available that can accurately predict Ab–Ag interactions. Lim *et al*. [16] developed a convolutional neural network (CNN) that uses complementarity-determining region (CDR) sequence characteristics to predict interactions between PD-1 and CTLA-4 Abs. Wang *et al*. [17] developed a model that utilizes a multi-head attention network with position embeddings of CDRs to accurately differentiate between Abs associated with influenza HA and the SARS-CoV-2 S protein. Ye *et al*. [18] also predicted Ab–Ag binding using random forest and weighted nearest-neighbor models based on sequencing data. Huang *et al*. [19] introduced AbAgIntPre, an online tool for predicting Ab–Ag interactions based on sequence features. AbAgIntPre used a CNN combined with the encoding of spaced amino acid pair (CKSAAP) composition. Pittala *et al*. [20] developed a comprehensive deep-learning framework to predict binding interfaces between Ags and Abs. Their approach integrates graph convolution networks, attention mechanisms, and transfer learning to capture key characteristics of Ab–Ag interactions effectively. Wang [21] employed a transformer-based neural network trained on DMS data to predict the escape percentages of Ab sequences against SARS-CoV-2 RBDs.

Despite the discovery of Nbs more than three decades ago [22], there needs to be more focus on gathering data and advancing computational methodologies tailored explicitly for handling these small molecules [23]. As highlighted in previous studies [24–29], it is imperative to leverage deep-learning techniques that address the diverse sequence, structural, and Ag-binding attributes of Nbs to facilitate their computational design. Modeling and predicting Nb structures remains challenging [30, 31]. Although the Protein Data Bank contains hundreds of crystallographic structures of Nbs [32, 33], current models fail to comprehensively depict the significant diversity in both structure and sequence observed in Nb hypervariable loops. Furthermore, Nbs exhibit more substantial variation in conformation, length, and sequence diversity in their CDR3 region compared to Abs [27], making it more difficult to understand and predict their 3D structures.

For accurate prediction of Nb structures, Tomer *et al*. [30] developed a sophisticated deep learning model called Nanonet. Nanonet utilizes a CNN of two 1D residual networks (ResNet) to perform 3D structural modeling of Nbs. Tam *et al*. [34] proposed a framework for predicting the posture of Nbs. They used modified parent poses to generate a set of features, which were then fed into a decision tree binary classifier. Additionally, Sardar *et al*. [35] employed various machine learning algorithms, including Support Vector Machine (SVM), K-Nearest Neighbors (KNN), Naive Bayes (NB), Multi-Layer Perceptron (MLP), Logistic Regression (LR), Random Forest (RF), and Decision Tree (DT) algorithms. These models employed gapped k-mers as an embedding technique to accurately predict binding interactions based solely on NANOBODY® and Ag sequences.

An antibody’s CDRs exhibit high conservation between the heavy and light immunoglobulin chains, crucial in facilitating Ag binding. As previously mentioned, numerous computational methodologies have been devised to predict Ab–Ag interactions based on either structure or sequence. While these methodologies have proven effective in predicting Ab–Ag interactions, they do not apply to Nbs [31] due to their reliance on information from both Ab chains, whereas Nbs possess only a heavy chain. Currently, a model based on classical machine learning methods, as described by Sardar *et al*. [35], exists for predicting the binding between Nbs and Ags. However, most recent work on biomolecular interaction prediction has shifted towards deep learning models. Therefore, we developed a deep learning model based on BERT to predict the interaction between Nbs and Ags.

## Methods and materials

### Datasets and preprocessing

The proposed model is trained using three distinct databases. The UNIPROT database is used for pretraining, while the NANOBODY®-Ag and binary PPI datasets are used for finetuning. The datasets are presented in the order of preprocessing and their interdependencies. Since the BERT model accommodates a maximum of 512 tokens, with three tokens reserved for the beginning, end, and separator between sequences, a total of 509 tokens is allowed for the two sequences combined. Table 1 shows the number of samples in the training and test datasets. Below is a concise description of the collected datasets. More details about preprocessing the datasets can be found in the Supplementary Material.

**Table 1:** Number of Samples for PPI and Nb-Ag datasets

Dataset	Training dataset		Test dataset	
	Pos set	Neg set	Pos set	Neg set
Nb-Ag	506	676	56	76
PPI	31556	31556	281	281

#### NANOBODY®-Ag dataset

We used the dataset curated by Sardar *et al*. [35], which contains 47 Ag sequences from UNIPROT [36]. For each Ag, we included all binding Nbs from the sdAb database [37], totaling 365 Nbs. We preprocessed the data using the following rules:

1. Selecting the binding and non-binding pairs using the Clustal Omega method to calculate the sequences’ proximity matrix.
2. Removing pairs whose combined token count exceeds 509 tokens when using a k-mer representation with k set to 3.

After preprocessing, 1314 pairs remained: 562 positive and 752 negative pairs. The final Nb-Ag dataset was divided into two subsets. The first subset is the training set, which includes 1182 pairs: 506 positive and 676 negative pairs, constituting 90% of the dataset. Within the training set, 5% is reserved for validation. The second subset is the test set comprising 132 pairs: 56 positive and 76 negative pairs, making up the remaining 10% of the total dataset. The Ag sequences comprise 13 unique protein sequences sourced from the UNIPROT database.

#### P‌PI data

The binary PPI dataset was sourced from the HINT database [38]. As of November 2023, this database contains positively interacting protein-protein (PP) pairs for 12 organisms. The dataset was prepared for training as follows:

1. Downloading and combining the binary interaction data for the 12 organisms.
2. To avoid redundancy in finetuning with PP data, remove the unique protein sequences of the Ags from the PP dataset.
3. Eliminating pairs whose combined token count exceeds 509 tokens while using a k-mer representation with k set to 3.
4. Dividing the interacting pairs into training and test sets.
5. Employing BLASTp [39] to eliminate homologous proteins in both the test and training datasets with a similarity threshold of 40%.
6. Generating random negative samples to maintain a 1:1 ratio of positive to negative cases.

After preparing the PP data, a training dataset consisting of 63,112 examples was created, with 5% of the training data reserved for validation. Additionally, a test dataset comprising 562 examples was created.

#### UNIPROT database

The protein sequences were downloaded from the UNIPROT database [36]. The entries were filtered based on the following rules:

1. Removing sequences with fewer than 100 tokens or more than 509 tokens when using a k-mer representation with $k$=3.
2. Removing protein sequences already present in the PPI dataset.

After filtering, the total number of entries used for pretraining is 798,860.

### NABP-BERT model structure

**Figure 1:**
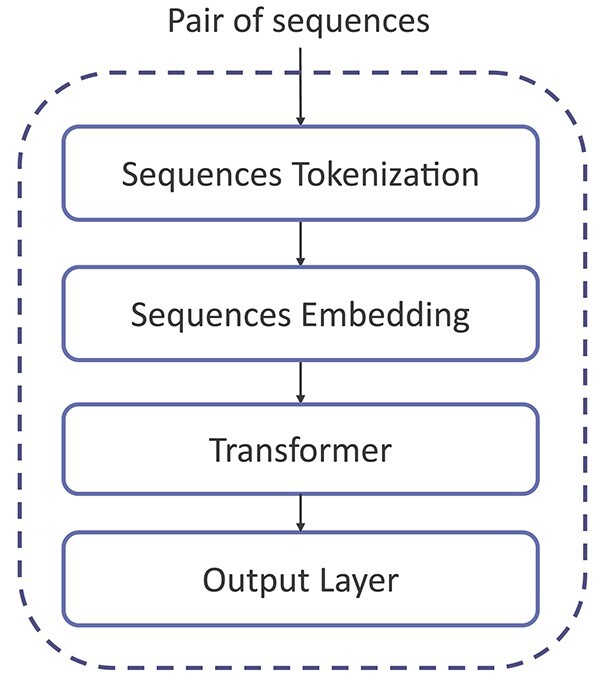
Structure of the BERT model consists of sequence tokenization, sequence embedding, transformer, and output layer.

This paper introduces a novel NABP-BERT model, which provides a comprehensive and transferable framework for predicting Nb-Ag interactions, focusing on the surrounding amino acid contexts. The NABP-BERT model uses the same structure as the BERT model, with some changes in the transformer module parameters. Figure 1 illustrates the complete structure of the BERT model, which consists of sequence tokenization, sequence embedding, transformer, and an output layer. A detailed description of the model structure is provided below.

#### Sequence tokenization

Nanobodies and Ags are composed of amino acid sequences, each represented by 20 distinct letters corresponding to the 20 natural amino acids. In this study, the NANOBODY® sequence is denoted as $N = [n_{1}, n_{2},..., n_{k}]$, and the Ag sequence as $A = [a_{1}, a_{2},....., a_{m}]$, where $n_{i}$ and $a_{i}$ are the $i$^th^ amino acids in the NANOBODY® and Ag sequences, respectively, and $k$ and $m$ represent the total number of amino acids in each sequence. Each pair of sequences in the Nb-Ag dataset used for finetuning is associated with a label $y$, where $y=1$ indicates that the NANOBODY® binds to the Ag, and $y=0$ indicates no binding.

**Figure 2:**
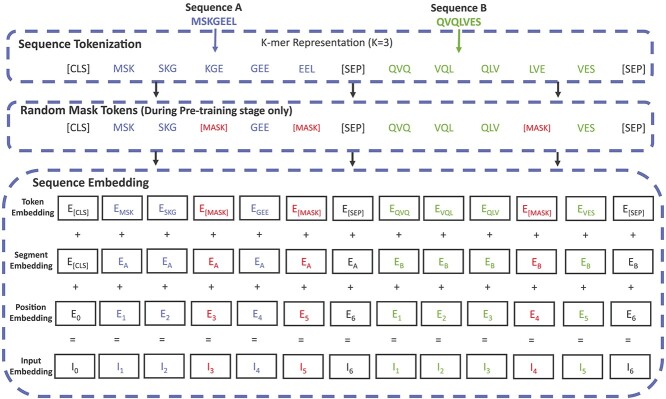
An example of sequence tokenization and embedding. The length of the NANOBODY® sequences and Ag sequences in this figure is an example.

Initially, we tokenize the input sequence using a k-mer representation, where each group of $k$ amino acids is treated as an individual token. This approach is commonly used in protein sequence analysis as it incorporates additional contextual information by combining each amino acid with its preceding ones. We adopt the same value of $k$ based on prior research [40], which demonstrated that setting $k$ to 3 achieves optimal performance. Consequently, the proposed model encodes the sequence using overlapping 3-mers of amino acids as tokens. For example, Fig. 2 illustrates how the protein sequence ’MSKGEEL’ can be tokenized into a sequence of five 3-mers: MSK, SKG, KGE, GEE, EEL. The vocabulary for $k=3$ consists of all the permutations of 3-mers plus four unique tokens: [CLS] for the classification token, [UNK] for the unknown token, [SEP] for the separation token, and [MASK] for the masked token. Thus, the total vocabulary size is $20^{3} + 4$, where 20 is the standard number of amino acids.

#### Sequence embedding

The input vector for each token in the model consists of three components: token embedding, segment embedding, and position embedding, as illustrated in Fig. 2. The token embedding encodes each 3-mer (three amino acids) based on a predefined vocabulary. The segment embedding distinguishes whether the token belongs to the first or second sequence in the pair. A positional encoding vector is integrated into the embedding vector to enable the model to capture the relative positional information of tokens within a sequence. The positional encoding vector is significant because the model does not employ recurrent or convolutional layers.

#### Transformer

**Figure 3:**
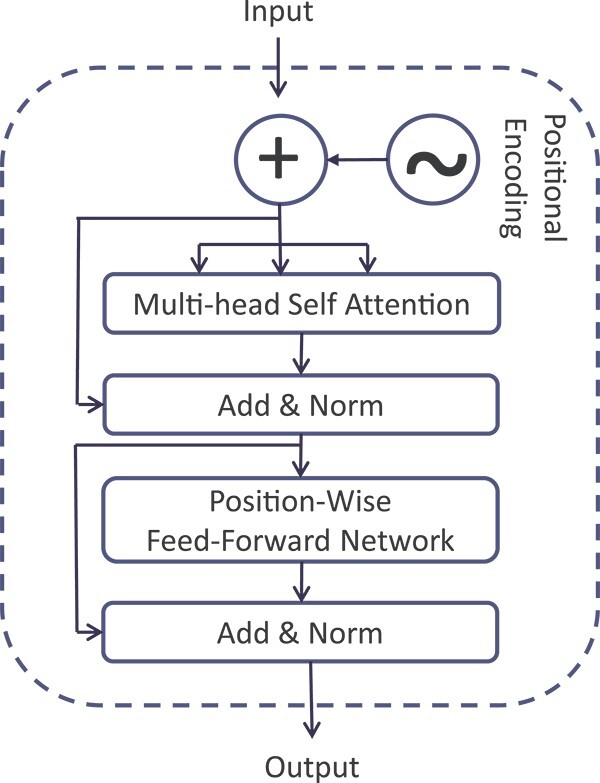
Structure of the transformer module

The input embedding is fed into the transformer architecture, as shown in Fig. 3. This transformer comprises multiple encoder layers containing positional encoding, multi-head self-attention (MSA), and a position-wise feed-forward network (PW-FFN). The PW-FFN consists of two fully connected layers with a ReLU activation function in between. The MSA and the PW-FFN are augmented with a residual connection, followed by a normalization layer, as described in [41]. Residual connections are employed to mitigate the vanishing gradient problem in deep networks. A comprehensive explanation of the transformer mechanism is provided in the work by Vaswani *et al*. Below is a concise explanation of the functioning of a transformer. Given an input sequence $X = (x_{1}, x_{2},..., x_{N})$, the output sequence of the self-attention head $H = (h_{1}, h_{2},..., h_{N})$ may be computed as follows:


(1) \begin{align*}& h_{i} = A\left(X,W^{Q},W^{K},W^{V}\right) = \sum_{j=1}^{N}a_{ij}\left(X,W^{Q},W^{K}\right)\left(x_{j}W^{V}\right)\end{align*}

where $x_{j}\in R^{d_{x}}$, $h_{i}\in R^{d_{h}}$, $W^{Q}, W^{K}, W^{V}\in R^{d_{x} \times d_{h}}$ are the parameter projection matrices. The weight coefficient $a_{ij}$ is computed using a softmax function:


(2) \begin{align*}& a_{ij}\left( X,W^{Q},W^{K} \right)=\frac{exp\left( \eta_{ij} \right)}{\sum_{k=1}^{N}exp\left( \eta_{ik} \right)}\end{align*}

The value of $\eta _{ij}$ is calculated using scaled dot-product attention:


(3) \begin{align*}& \eta_{ij}=\frac{\left( x_{i}W^{Q}\right)\left(x_{j}W^{K}\right)^{T}}{\sqrt{d_{h}}}\end{align*}

The MSA utilizes self-attention multiple times to focus on information from various representation subspaces at different positions simultaneously. This is formally defined as follows:


(4) \begin{align*}& MSA\left( x \right) = Concat\left( H_{1}, H_{2},..., H_{\Omega} \right)W^{O}\end{align*}

The symbol *Concat* denotes the concatenation operation, ${\Omega }$ represents the number of heads, and $W^{O} \in R^{\Omega d_{x} \times d_{h}}$ is a matrix used for projection. In our study, the transformer module is configured with one encoder stack, eight MSA heads, a PW-FFN with 3072 hidden units, and a transformer hidden size of 768.

#### Output layer

At the output, the [CLS] representation is fed into an output layer for classification, such as next sentence prediction, protein-protein interaction prediction, or NANOBODY®-Ag binding prediction.

### Model training

The proposed model adopts a pretraining-finetuning strategy. In the pretraining stage, self-supervised learning is employed to capture the general representation of protein sequences. The finetuning stage then uses supervised learning to learn the relationships between pairs of sequences. Initially, the proposed model accepts input sequences represented by k-mer tokens. This input representation effectively captures single and paired sentences within a unified token sequence, enabling our model to perform well in diverse downstream tasks. Specifically, for tasks involving PPI and NABP, the input sequence is structured as a pair of sentences: one representing ProteinA/NANOBODY® and the other representing ProteinB/Antigen. These two sentences are combined into a single sequence. The initial token in each sequence is consistently a unique classification token, denoted as [CLS]. The final hidden state corresponding to this token is the comprehensive representation of the entire sequence for classification purposes.

We distinguish between the two sentences in two ways: first, by using a unique separator token [SEP], and second, by adding a segment embedding to each token to indicate whether it belongs to sentence A (ProteinA/NANOBODY®) or sentence B (ProteinB/Antigen). After tokenizing the sequence, a masking strategy is applied, and the sequence is then passed to the embedding layer. It is worth noting that the masking strategy is applied only during the pretraining stage. The tokenized sequence is directly fed into the embedding layer during the finetuning stage.

#### Self-supervised pretraining

**Figure 4:**
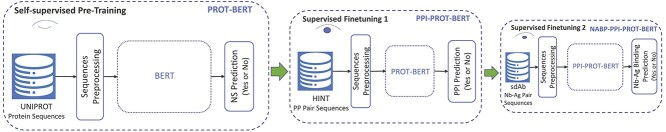
The training process of the proposed NABP-PPI-PROT-BERT model consists of three stages: self-supervised pretraining, supervised finetuning 1, and supervised finetuning 2.

During the pretraining phase, PROT-BERT learns the fundamental syntax and semantics of protein sequence data from the UNIPROT database. It utilizes self-supervised learning in the general pretraining stage for 1 000 000 epochs, as depicted in Fig. 4. PROT-BERT is pretrained using two unsupervised tasks on unlabeled data.

The first task is the Masked Language Model (MLM). In this task, a certain percentage of input tokens (3-mer amino acids) are randomly masked in a 512-length sequence, and the model is trained to predict these masked tokens to develop a deep bidirectional representation. The final hidden vectors for the masked tokens are passed through a Softmax output layer across the vocabulary. The Supplementary Material provides more details about the MLM task throughout our experiments.

The second task is Next Sentence Prediction, which is crucial for downstream tasks that require understanding the relationship between two sentences. This relationship is not directly addressed by traditional language modeling, as seen in tasks such as predicting PPIs and Nb-Ag binding. To enable the model to comprehend the connections between sentences, we pre-train it to predict the subsequent sentence. For each pretraining example, when selecting sentences A and B, B is chosen as the immediate subsequent sentence to A (labeled as IsNext) 50% of the time, and as a random sentence from the corpus (labeled as NotNext) the remaining 50% of the time.

#### Supervised finetuning

The PROT-BERT model undergoes two levels of finetuning following pretraining, as illustrated in Fig. 4. In the first level, the model is finetuned to predict interactions between annotated PP pairs. The model refined at this level is referred to as the PPI-PROT-BERT model. In the second level, the PPI-PROT-BERT model is finetuned using labeled data from Nb-Ag pair sequences to predict the binding between NANOBODY® and Ag sequences. The model developed at this stage is denoted as the NABP-PPI-PROT-BERT model. Each finetuning level is trained 10 times, each training session consisting of 100 epochs. The results presented in this paper represent the average performance across these 10 executions.

### Implementation details

The model was trained using a mini-batch size of 8 samples with back-propagation and binary cross-entropy loss. The Adam optimizer with accelerated adaptive moment estimation was used to minimize the loss, with the learning rate set to $2\times 10^{-5}$. The training was conducted on a server equipped with a GeForce GTX 2080 Ti GPU with 11 GB of VRAM and a total memory of 251 GB. Details of the software libraries and framework versions are provided in the ’requirements.txt’ file, which is available alongside the code in the GitHub repository.

## Results and discussion

### Performance Evaluation by Varying the Number of Attention Heads

We evaluated the performance of NABP-PPI-PROT-BERT by varying the number of attention heads in the transformer module. The number of attention heads can only be adjusted if the hidden size of the transformer is divisible by the number of attention heads. Given that the hidden size is 768, we configured the number of attention heads to be 1, 2, 3, 4, 6, 8, and 12. Throughout these experiments, the number of encoder layers was fixed at 1. Table 2 presents the results of the NABP-PPI-PROT-BERT model with different numbers of attention heads, evaluated using AUROC and AUPR metrics. Notably, the performance across all experiments was satisfactory, with AUROC and AUPR values exceeding 0.96. The optimal performance was achieved with 8 attention heads, resulting in AUROC and AUPR values of 0.986 and 0.985, respectively.

**Table 2:** The performance of the NABP-PPI-PROT-BERT model by varying the number of attention heads with a single encoder in terms of AUROC and AUPR.

# attention heads	AUROC	AUPR
1	0.968	0.967
2	0.982	0.976
3	0.964	0.966
4	0.973	0.972
6	0.978	0.978
**8**	**0.986**	**0.985**
12	0.971	0.973

Note: the best performance is given in **boldface**.

### Performance evaluation by varying the number of encoder layers

In this experiment, the performance of NABP-PPI-PROT-BERT was evaluated by varying the number of encoder layers in the transformer module. The encoder layers were tested with configurations of 1, 2, 4, 6, 8, 10, and 12 layers, while the number of attention heads was kept constant at 8 across all experiments. Table 3 shows the performance of the NABP-PPI-PROT-BERT model with different numbers of encoder layers, as measured by AUROC and AUPR metrics. All configurations delivered satisfactory results, with AUROC and AUPR values exceeding 0.92. However, it is essential to note that the model’s performance tends to decrease as the number of encoder layers increases. Specifically, the AUROC dropped from 0.986 to 0.921, and the AUPR decreased from 0.985 to 0.927 as the number of encoder layers increased from 1 to 12. Thus, our model suggests that a shallow BERT network, comprising a single encoder layer and eight attention heads, is sufficient for the NABP task.

**Table 3:** The performance of the NABP-PPI-PROT-BERT model by varying the number of encoder layers and number of attention heads is set to 8 in terms of AUROC and AUPR.

# encoder layers	AUROC	AUPR
**1**	**0.986**	**0.985**
2	0.974	0.972
4	0.960	0.964
6	0.959	0.963
8	0.948	0.949
10	0.927	0.930
12	0.921	0.927

Note: the best performance is given in **boldface**.

### Evaluation of performance through finetuning the pretrained PROT-BERT model with NANOBODY®-Ag Data

**Figure 5:**
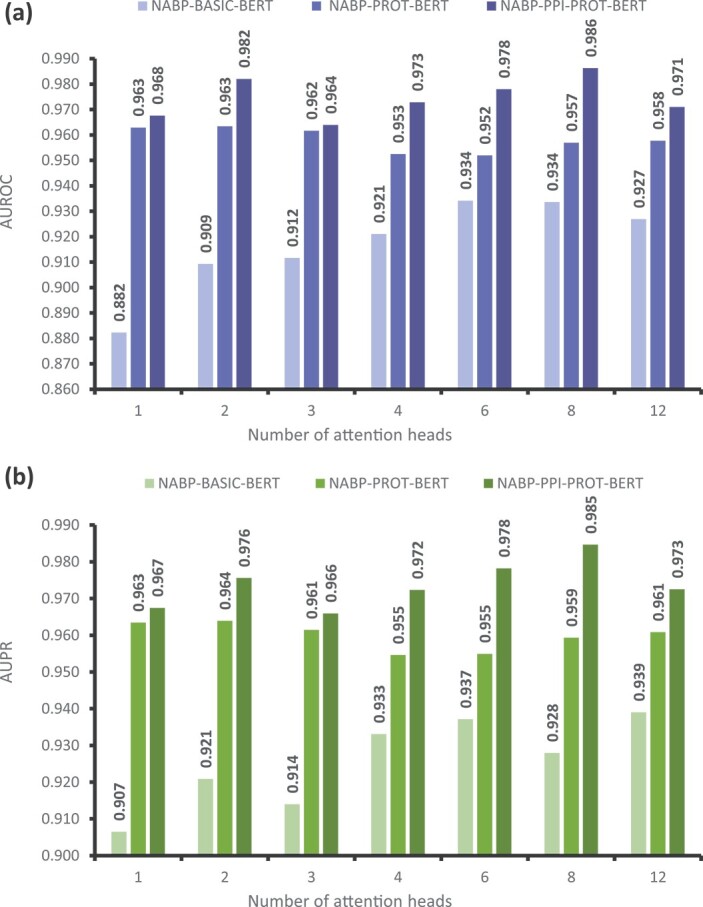
Comparison between NABP-BASIC-BERT, NABP-PROT-BERT, and NABP-PPI-PROT-BERT models by varying the number of attention heads with a single encoder, in terms of (a) AUROC, (b) AUPR.

**Figure 6:**
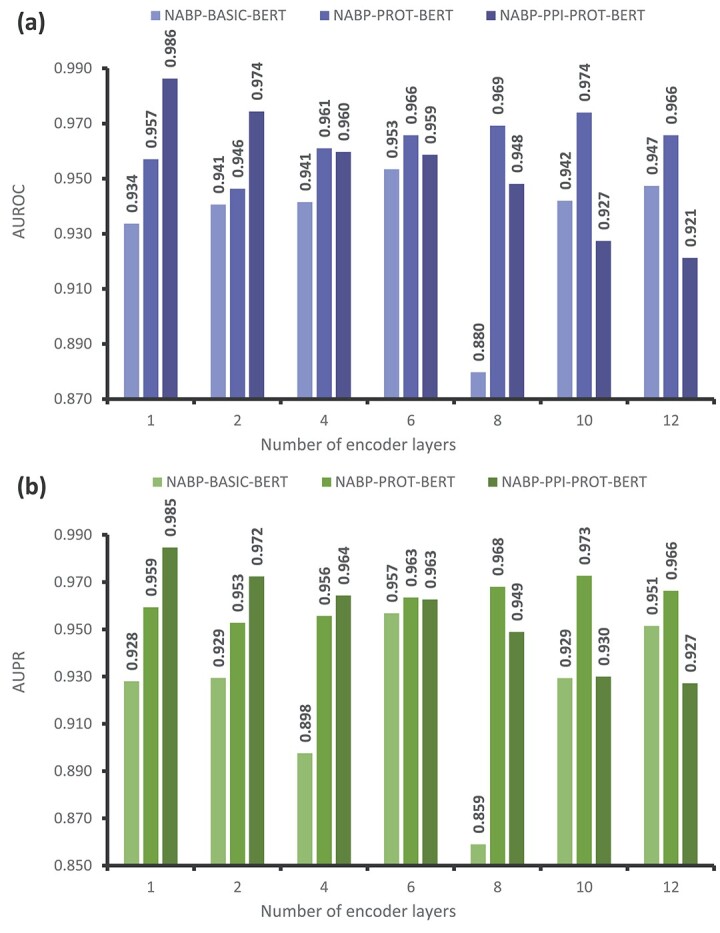
Comparison between NABP-BASIC-BERT, NABP-PROT-BERT, and NABP-PPI-PROT-BERT models by varying the numbers of encoder layers, and the number of attention heads is fixed at 8, in terms of (a) AUROC, (b) AUPR.

Further experiments were conducted to optimize the pretrained ’PROT-BERT’ model using Nb-Ag data. This refined model, named NABP-PROT-BERT, underwent a single round of finetuning. Figures 5 and 6 compare the performance of NABP-PROT-BERT with NABP-PPI-PROT-BERT, focusing on different numbers of attention heads and encoder layers, respectively. Figure 5 demonstrates that the NABP-PPI-PROT-BERT model outperforms the NABP-PROT-BERT model in terms of both AUROC and AUPR metrics across all attention head values when using a shallow BERT network with a single encoder. The NABP-PPI-PROT-BERT model shows superior improvements of 0.47%, 1.87%, 0.23%, 2.04%, 2.60%, 2.93%, and 1.33% in AUROC, and 0.40%, 1.16%, 0.45%, 1.77%, 2.33%, 2.53%, and 1.17% in AUPR, at attention head values of 1, 2, 3, 4, 6, 8, and 12, respectively.

Figure 6 compares the two proposed models with varying numbers of encoder layers while keeping the number of attention heads fixed at 8. When using two or four encoders, the performance of both models is similar to that with a single encoder. However, with six encoders, which constitute a deeper BERT network, NABP-PROT-BERT outperforms NABP-PPI-PROT-BERT in terms of AUROC, showing improvements of 0.71%, 2.11%, 4.66%, and 4.45% at encoder layers 6, 8, 10, and 12, respectively. In terms of AUPR, NABP-PROT-BERT outperforms NABP-PPI-PROT-BERT by 0.08%, 1.91%, 4.27%, and 3.91% at encoder layers 6, 8, 10, and 12, respectively.

The most optimal results were achieved with a shallow BERT network using a single encoder and eight attention heads, combined with two rounds of finetuning. The NABP-PPI-PROT-BERT model achieved AUROC and AUPR values of 0.986 and 0.985, respectively. In contrast, a deep BERT network with 10 encoders and 8 attention heads, with a single round of finetuning, yielded AUROC and AUPR values of 0.974 and 0.973 for NABP-PROT-BERT. Therefore, a shallow BERT network with dual finetuning levels performs better than a deep network with a single finetuning level. However, constructing the NABP-PPI-PROT-BERT model requires two finetuning levels, making it significantly more time-consuming than the single finetuning required for NABP-PROT-BERT. This time difference is notable, assuming both models have identical architectures, including the same number of encoder layers and attention heads.

### Effect of using the self-supervised pretraining stage

This experiment investigates the impact of the pretraining mechanism on the predictive performance of NABP recognition models. For each model architecture, where the number of attention heads or encoders is varied, we bypassed the pretraining phase and directly trained the models using the Nb-Ag pairs dataset. The resulting model is referred to as NABP-BASIC-BERT. Figures 5 and 6 present the experimental results of the NABP-BASIC-BERT model compared to the NABP-PROT-BERT and NABP-PPI-PROT-BERT models in terms of AUROC and AUPR. There is a significant enhancement in evaluation metrics due to pertaining, as shown in Figures 5 and 6. This confirms the effectiveness of pretraining in acquiring knowledge about the relationships between amino acids by identifying shared characteristics in protein sequences. As a result, it enhances the model’s capability to identify NABP in subsequent tasks.

When a single encoder is used with varying numbers of attention heads, the impact of pretraining on improving model performance becomes more apparent. The NABP-PROT-BERT models show improvements of 8.07%, 5.41%, 5.00%, 3.14%, 1.78%, 2.34%, and 3.09% in AUROC for attention heads 1, 2, 3, 4, 6, 8, and 12, respectively. In terms of AUPR, the improvements are 5.69%, 4.31%, 4.74%, 2.15%, 1.78%, 3.14%, and 2.18% over the NABP-BASIC-BERT models with the same attention heads. The NABP-PPI-PROT-BERT model demonstrates improvements of 8.54%, 7.28%, 5.23%, 5.18%, 4.38%, 5.27%, and 4.41% in AUROC, and 6.10%, 5.47%, 5.20%, 3.92%, 4.11%, 5.67%, and 3.35% in AUPR, compared to the NABP-BASIC-BERT models at attention heads 1, 2, 3, 4, 6, 8, and 12, respectively, as shown in Fig. 5.

Figure 6 illustrates that when using various numbers of encoder layers with eight attention heads, the NABP-PROT-BERT model achieves the following improvements: 2.34%, 0.58%, 1.95%, 1.2%, 8.95%, 3.20%, and 1.83% in AUROC. Similarly, in terms of AUPR, improvements of 3.14%, 2.33%, 5.81%, 0.66%, 10.90%, 4.33%, and 1.48% are observed over the NABP-BASIC-BERT models at encoder layers of 1, 2, 4, 6, 8, 10, and 12, respectively. The NABP-PPI-PROT-BERT models demonstrate improvements of 5.27%, 3.38%, 1.82%, 0.52%, and 6.84% in AUROC. Similarly, in terms of AUPR, there are improvements of 5.67%, 4.30%, 6.68%, 0.59%, and 8.99% over the NABP-BASIC-BERT models at encoder layers 1, 2, 4, 6, and 8, respectively. However, with 10 and 12 encoder layers, there is a significant decrease in performance by 1.46% and 2.62% in AUROC, respectively. Regarding the AUPR metric, a drop of 2.43% is observed with 10 encoders. Adopting deep layers in the BERT network leads to a degradation in performance, which is not advantageous when dealing with the NABP problem. By pretraining on a substantial amount of protein samples, it becomes feasible to effectively capture the relationships between amino acid molecules in protein sequences, thereby reducing the learning burden on the model. Pretraining is essential for optimizing the performance of the model.

### Performance comparison with state-of-the-art methods

This study aims to develop a deep learning approach for predicting Nb-Ag binding based solely on sequence information. We found only one prior study [35] that explored this task exclusively using sequence data. Several classical machine learning algorithms were employed in that study, including SVM, NB, MLP, KNN, RF, LR, and DT. To ensure a fair comparison, we re-implemented these machine learning models using the same Nb-Ag dataset used for the proposed models. Additionally, we maintained the same training-to-test data ratio and used the same performance metrics, AUROC and AUPR. We then compared the proposed deep learning models—NABP-BASIC-BERT, NABP-PROT-BERT, and NABP-PPI-PROT-BERT—and the re-implemented models suggested by Sardar *et al*. [35].

Table 4 presents the performance of the NABP-BASIC-BERT, NABP-PROT-BERT, and NABP-PPI-PROT-BERT models, as well as the re-implemented ML models, in terms of AUROC and AUPR. The NABP-BASIC-BERT model is built with 6 encoders and 8 attention heads, the NABP-PROT-BERT model comprises 10 encoders with 8 attention heads, and the NABP-PPI-PROT-BERT model consists of a single encoder with 8 attention heads. We focus on these three models as they exhibit superior performance compared to the other proposed models. The KNN method outperforms all other traditional ML algorithms, achieving AUROC and AUPR scores of 0.933 and 0.899, respectively. In contrast, the NABP-BASIC-BERT, NABP-PROT-BERT, and NABP-PPI-PROT-BERT models outperform it, achieving AUROC and AUPR scores of 0.953 and 0.957, 0.974 and 0.973, and 0.986 and 0.985, respectively.

**Table 4:** Comparison between NABP-BASIC-BERT, NABP-PROT-BERT, and NABP-PPI-PROT-BERT models and state-of-the-art methods in terms of AUROC and AUPR.

Model	AUROC	AUPR
**NABP-PPI-PROT-BERT**	**0.986**	**0.985**
**NABP-PROT-BERT**	**0.974**	**0.973**
**NABP-BASIC-BERT**	**0.953**	**0.957**
SVM	0.793	0.728
NB	0.846	0.789
MLP	0.635	0.586
KNN	0.933	0.899
RF	0.909	0.887
LR	0.826	0.766
DT	0.907	0.868

Note: the performance of better models is in **Boldface**, and the best one is underlined.

These results demonstrate that deep learning algorithms yield superior performance to classical ML algorithms. Specifically, the NABP-BASIC-BERT model shows improvements of 2% and 5.8% in AUROC and AUPR, respectively, compared to the KNN algorithm. The NABP-PROT-BERT model demonstrates enhancements of 4.1% and 7.4% in AUROC and AUPR, respectively, compared to KNN. Finally, the NABP-PPI-PROT-BERT model exhibits improvements of 5.3% and 8.6% in AUROC and AUPR, respectively, over the KNN algorithm. Among these models, NABP-PPI-PROT-BERT emerges as the most superior.

## Conclusion

This paper introduces a novel sequence-based deep learning model leveraging the BERT architecture to predict NANOBODY®-Ag interactions, thereby reducing the need for computationally intensive techniques such as docking. The proposed models employ a pretraining-finetuning approach. During the pretraining phase, the model efficiently captures information regarding the relationships between amino acids in protein sequences. The finetuning process comprises two stages: finetuning with protein-protein pair data and finetuning with Nb-Ag pair data. Experimental results reveal that employing a shallow BERT network with two levels of finetuning yields superior performance compared to utilizing a deep network with a single level of finetuning. Our proposed NABP-BERT models outperform other state-of-the-art methods in predicting NANOBODY®-Ag binding. Furthermore, a comprehensive pretrained model, trained on a large dataset of protein sequences, can be used for subsequent protein-related tasks. This versatile model, trained on protein-protein pair sequences, can be adopted for downstream protein-protein tasks. Specifically, when the structure of a NANOBODY®’s binding site is not accessible, the proposed NABP-BERT method, which is publicly accessible, will be a powerful tool in the expanding field of NANOBODY®-Ag binding prediction. Moreover, it can accelerate the progress of Nb-based diagnostics and therapies for various diseases, such as cancer and other severe illnesses. Our future focus will be on utilizing other deep learning algorithms and extracting additional structural features from NANOBODY® and Ag sequences to enhance the performance of our model.

**Key Points**

- Developed the PROT-BERT model as a general model for various protein-related tasks.
- Introduced the PPI-PROT-BERT model as a base model for protein-protein interaction tasks.
- Proposed the NABP-PROT-BERT and NABP-PPI-PROT-BERT models specifically designed for predicting NANOBODY®-antigen binding.
- Demonstrated that our proposed models outperform existing state-of-the-art methods.

## Supplementary Material

Supplementary_Material_bbae518

## Availability of data and code

The source code, models, and data are available on https://github.com/FMoonlightS/NABP-BERT.
