# Supplementary material for: NABP-BERT: NANOBODY®-antigen binding prediction based on bidirectional encoder representations from transformers (BERT) architecture
Source: Brief Bioinform. 2024 Dec 17;26(1):bbae518. doi: 10.1093/bib/bbae518 (PMC11650500; doi:10.1093/bib/bbae518)
Supplement: Supplementary_Material_bbae518 [file supplementary_material_bbae518.pdf]

# Supplementary Material

## NABP-BERT: NANOBODY®-antigen binding prediction based on bidirectional encoder representations from transformers (BERT) architecture

### Datasets and Preprocessing

The proposed model is trained using three distinct databases. The UNIPROT database is employed for pretraining the model, while the NANOBODY®-Antigen and binary PPI datasets are utilized for finetuning. The following are the details of the collected datasets. The datasets are presented in the order of preprocessing and their interdependencies.

### NANOBODY®-Antigen Dataset

We employed the dataset curated by Sardar et al. [35], which contains 47 Antigen (Ag) sequences from UNIPROT [36]. We included all binding nanobodies (Nbs) for each antigen from the single domain antibody database [37], totaling 365 nanobodies. Binding and non-binding pairs are selected using the method employed in Clustal Omega to calculate the proximity matrix for these sequences. This matrix evaluates the pairwise edit distance between antigens. The pairwise distance is utilized to identify Nb-Ag pairs that bind or non-bind, depending on specified threshold values. We used the same threshold values as those in Sardar et al.'s work [35]. If there are two binding pairs ($n_i$, $g_j$) and ($n_l$, $g_k$), then binding pairs are possible when the edit distance between $g_j$ and $g_k$ is less than 0.2, i.e.; ($n_l$, $g_j$) and ($n_i$, $g_k$) are considered binding pairs. As a result, 1388 additional binding pairs are obtained; thus, the total number of binding pairs is 1753. Conversely, any pair with a distance between $g_j$ and $g_k$ greater than 0.85 can be designated a non-binding pair. We randomly selected 1753 pairs to represent the non-binding pairs.

The maximum length of the nanobody and antigen sequences is 175 and 1816, respectively. Given that the BERT model is designed to accommodate a maximum of 512 tokens, including three tokens reserved for the beginning, end, and separator between the two sequences, a total of 509 tokens are allowed for the combined sequences. Consequently, the nanobody and antigen sequences are filtered based on their lengths to ensure their combined token count does not exceed 509 tokens when using a k-mer representation with k set to 3. After this length-based filtering, 1314 pairs remain, with 562 positive pairs and 752 negative pairs. After cleaning, the antigen sequences comprise 13 unique protein sequences sourced from the UNIPROT database. The final Nb-Ag dataset was divided into two subsets. The first subset is the training set, which includes 1182 pairs: 506 positive pairs and 676 negative pairs, constituting 90% of the dataset. Within the training set, 5% is reserved for validation. The second subset is the test set, comprising 132 pairs: 56 positive pairs and 76 negative pairs, making up the remaining 10% of the total dataset.

## PPI Data

The binary PPI dataset was sourced from the HINT database [38]. As of November 2023, this database encompasses PPI data for 12 organisms: H. sapiens, S. cerevisiae, S. pombe, M. musculus, D. melanogaster, C. elegans, A. thaliana, B. subtilis, B. taurus, E. coli, R. norvegicus, and O. sativa. The dataset was prepared for training as follows: binary interaction data, which includes the protein pairs that interact positively, were downloaded for 12 organisms and consolidated. This yielded 226,834 interaction pairs involving 47,725 unique proteins. To avoid redundancy in finetuning with protein-protein data, the protein sequences corresponding to the Nb-Ag pairs present in the PPI data were excluded from the unique proteins in the dataset, resulting in 47,718 unique proteins. The protein sequences were obtained from a local version of the Swiss-Prot and Trembl databases [36] where accessible. A total of 46,314 protein sequences (97.06%) were successfully retrieved from the databases. Protein pairs with a combined length exceeding 509 tokens were excluded from the PPI database. After filtration, the database comprised 35,063 interaction pairs involving 13,647 distinct proteins.

The interacting pairs were divided into a training set comprising 31,556 (90%) and a test set comprising 3,507 (10%). To validate the models' accuracy on the test data, we excluded any proteins in the test dataset that shared more than 40% similarity with proteins in the training set. This ensured that the accuracy was based on a general pattern learned by the model rather than homology. BLASTp [39] was employed to eliminate homologous proteins in both the test and training datasets. Subsequently, the test proteins were compared against the protein sequences in the training dataset using BLAST (Basic Local Alignment Search Tool), and any test proteins exhibiting over 40% similarity were excluded from the final test set. This approach resulted in a test dataset comprising 281 interacting pairs. Random sampling was employed to generate negative examples and produce a dataset of equal size, maintaining a 1:1 ratio of positive to negative cases. The positive and negative pairs were merged to create a training dataset consisting of 63,112 examples, with 5% of the training data reserved for validation. Additionally, a test dataset was created, comprising 562 examples."

## UNIPROT database

The protein sequences were downloaded from the UNIPROT database [36], containing Swiss-Prot and Trembl databases with 570,419 and 251,131,638 entries, respectively. The entries of the Swiss-Prot and Trembl databases are filtered by the sequence length, where sequences with tokens less than 100 and larger than 509 are removed when using k-mer representation (k=3). After filtering, 409,275 entries were obtained, then the existing protein sequences in the PPI dataset were removed, so 399,430 entries were obtained. Because the Trembl database's entries are very large, 1,131,638 are selected randomly, and the same filtering rules are applied; as a result, 732,027 entries are obtained. Finally, we randomly select the same number of entries, such as Swiss-Prot, so the total entries used as pretrained data are 798,860.

**Self-supervised pretraining**

During the pretraining phase, PROT-BERT learns the fundamental syntax and semantics of protein sequence data from the UNIPROT database. It utilizes self-supervised learning in the general pretraining stage for 1,000,000 epochs. PROT-BERT is pretrained using two unsupervised tasks on unlabeled data.

The first task is the Masked Language Model (MLM). In this task, a certain percentage of input tokens (3-mer amino acids) are randomly masked in a 512-length sequence, and the model is trained to predict these masked tokens to develop a deep bidirectional representation. The final hidden vectors for the masked tokens are passed through a Softmax output layer across the vocabulary. Throughout our experiments, we randomly mask 15% of all k-mer tokens in each sequence, focusing solely on predicting the masked words rather than reconstructing the complete input. While this approach helps to obtain a pretrained bidirectional model, a drawback is a mismatch between the pretraining and finetuning stages, as the [MASK] token is absent during finetuning. We occasionally replace "masked" words with the literal [MASK] token to address this limitation. The training data generator randomly selects 15% of the token places for prediction. When selecting the $i^{th}$ token, it is replaced 80% of the time with the [MASK] token, 10% of the time with a random token, and 10% with the original $i^{th}$ token. The original token is estimated via cross-entropy loss by leveraging the final hidden vector of the $i^{th}$ input token.
